# Transcriptome Analysis of the Asian Honey Bee *Apis cerana cerana*

**DOI:** 10.1371/journal.pone.0047954

**Published:** 2012-10-24

**Authors:** Zi Long Wang, Ting Ting Liu, Zachary Y. Huang, Xiao Bo Wu, Wei Yu Yan, Zhi Jiang Zeng

**Affiliations:** 1 Honeybee Research Institute, Jiangxi Agricultural University, Nanchang, Jiangxi, China; 2 Department of Entomology, Michigan State University, East Lansing, Michigan, United States of America; 3 Department of Ecology, Evolutionary Biology and Behavior Program, Michigan State University, East Lansing, Michigan, United States of America; Boston University Medical Center, United States of America

## Abstract

**Background:**

The Eastern hive honey bee, *Apis cerana cerana* is a native and widely bred honey bee species in China. Molecular biology research about this honey bee species is scarce, and genomic information for *A. c. cerana* is not currently available. Transcriptome and expression profiling data for this species are therefore important resources needed to better understand the biological mechanisms of *A. c. cerana*. In this study, we obtained the transcriptome information of *A. c. cerana* by RNA-sequencing and compared gene expression differences between queens and workers of *A. c. cerana* by digital gene expression (DGE) analysis.

**Results:**

Using high-throughput Illumina RNA sequencing we obtained 51,581,510 clean reads corresponding to 4.64 Gb total nucleotides from a single run. These reads were assembled into 46,999 unigenes with a mean length of 676 bp. Based on a sequence similarity search against the five public databases (NR, Swissport, GO, COG, KEGG) with a cut-off E-value of 10^−5^ using BLASTX, a total of 24,630 unigenes were annotated with gene descriptions, gene ontology terms, or metabolic pathways. Using these transcriptome data as references we analyzed the gene expression differences between the queens and workers of *A. c. cerana* using a tag-based digital gene expression method. We obtained 5.96 and 5.66 million clean tags from the queen and worker samples, respectively. A total of 414 genes were differentially expressed between them, with 189 up-regulated and 225 down-regulated in queens.

**Conclusions:**

Our transcriptome data provide a comprehensive sequence resource for future *A. c. cerana* study, establishing an important public information platform for functional genomic studies in *A. c. cerana*. Furthermore, the DGE data provide comprehensive gene expression information for the queens and workers, which will facilitate our understanding of the molecular mechanisms of the different physiological aspects of the two castes.

## Introduction

*Apis cerana cerana*, the Eastern honey bee, is a honey bee species native to China. It is widely-kept in China, with more than 2 million colonies, and brings substantial economic benefits to beekeepers. Compared with the Western honey bee (*Apis mellifera*), *A. c. cerana* has a stronger resistance to the mite, *Varroa destructor* [1], and is better at collecting nectar from scattered floral resources [2].

Despite its economic importance, molecular biology research and sequence information for *A. c. cerana* functional genes are extremely lacking. Currently, there are only 124 mRNA sequences available in the NCBI database for *A. c. cerana*. This is extremely unfavorable for carrying out gene function research in *A. c. cerana*. Therefore, obtaining more gene transcription information of *A. c. cerana* is important for unraveling transcriptome complexity; for the identification of new transcription units, alternative splicing, and single nucleotide polymorphisms (SNP); and for performing gene function research in this species.

The queens and workers are two different castes of female honey bees with different physiological characteristics, such as body size, behavior, physiology, and life-span [3]. For example, the queens are fertile, while the workers are nearly sterile. Moreover, the life-span of queens is 10 times longer than that of the workers. Much research about caste differentiation and other physiological differences between queens and workers has been conducted in the Western honey bee, *A. mellifera* [4]–[19]. Severson *et* *al*. performed the first large-scale study on the molecular biology of caste differentiation in *A. mellifera* [4]. They demonstrated, by *in vitro* translation analyses that queens and workers differ in their mRNA profiles during the larval and prepupal stages. Evans & Wheeler first identified seven differentially expressed genes by subtractive hybridization [5]. Further research by microarray indicated that queens overexpressed several metabolic enzymes and workers overexpressed a member of the cytochrome P450 family, hexameric storage proteins, and dihydrodiol dehydrogenase [6]. Using a cDNA microarray based on more than 6,000 *A. mellifera* ESTs, Barchuk *et* *al*. found 240 differentially expressed genes (DEGs) between developing queens and workers [7]. They found that many DEGs are likely to be involved in processes favoring the development of caste-biased structures, such as brain, legs, and ovaries, as well as genes that code for cytoskeleton constituents. Other studies revealed gene expression differences between the two castes in the hypoxia pathway [8], TOR pathway [9], [10], insulin signaling pathway [11], [12], antioxidant pathway [13], and reproductive status [14]–[16]. Li *et* *al*. compared the expression differences of total proteins, mitochondrial proteins, and nuclear proteins during larvae caste determination of *A. mellifera* using proteomic approaches [17]–[19].

Aside from the honey bee, studies for analyzing gene expression differences between queens and workers have also been conducted in other social Hymenoptera insects, including Bumble bee *Bombus terrestris* [20], stingless bee *Melipona quadrifasciata* [21], [22] and ant *Lasius niger* [23].

Illumina RNA sequencing (RNA-seq) is a recently developed high-throughput sequencing method which uses deep sequencing technology to produce millions of short cDNA reads. By aligning these short reads against a reference genome, or assembling them *de novo* without the genomic sequence, we can quickly get a genome-scale transcription map containing both the transcriptional structure and the level of expression for each gene. This technology was widely used for creating *de novo* assembly in many organisms, including some insects (e.g. *Bemisia tabaci* [24] and *Nilaparvata lugens* [25]), and plants (e.g. *Taxus* [26] and *Hevea brasiliensis* [27]).

Digital gene expression (DGE), an improved version of the serial analysis of gene expression (SAGE) technique, is another recently developed approach for gene expression analysis. It is a tag-based transcriptome sequencing approach in which many 21 bp tag sequences from the 3′ end of each mRNA molecules are produced by high-throughput sequencing. The expression level of all the genes in a sample is measured by counting the number of tags produced from each gene. Compared with RNA-seq, DGE protocol is more suitable and affordable for comparative gene profiling without compromise or potential bias. DGE and RNA seq technologies have been used in transcriptome profiling studies for various applications, including cellular development, cancer, and immune defense in various organisms [28]–[30].

In this study, we constructed a cDNA library and obtained 46999 unigenes through Illumina RNA sequencing and sequence assembly. Moreover, we constructed two DGE libraries of the newly emerged queens and workers, and obtained 414 differentially expressed genes between them after sequencing and data analysis. All these results provide a shortcut for identifying new functional genes and useful information for studying the molecular biology of the queen-worker differentiation in *A. c. cerana*.

## Results

### Illumina sequencing and sequence assembly

**Figure 1:**
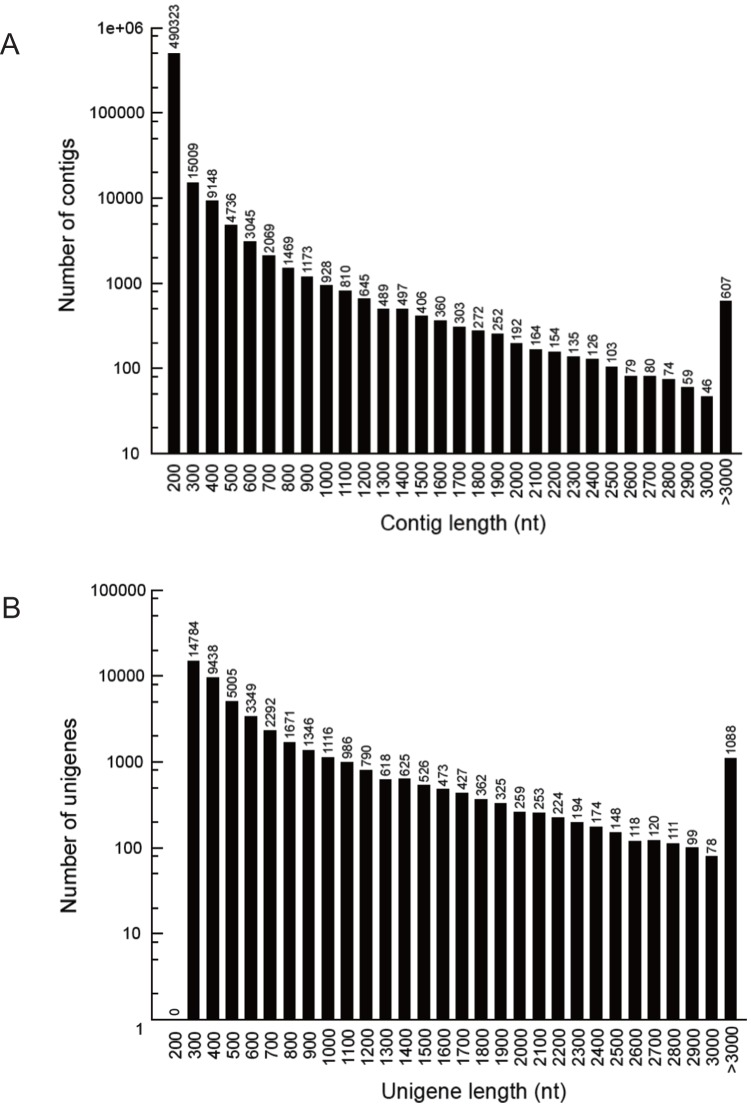
Length distributions of the *de novo* assembly for contigs and unigenes. The length distribution of contigs and unigenes were counted with an interval of every 100 bp from 200 bp to 3000 bp. Each number in the x-axis indicates a region of sequence length covering 100 bp, for example, “200”represents a region of sequence length [200, 300).

Illumina RNA sequencing generated a total of 55,303,332 raw reads (Table 1). After filtration, 51,581,510 clean reads with accumulated length of 4,642,335,900 bp remained for further analysis, the Q20 percentage (sequencing error rate, 1%) was 97.55%, and the GC percentage was 41.92%. These clean reads were assembled into 99,250 contigs with a mean length of 332 bp. The N50 of contigs was 518 bp. These contigs were further assembled by paired-end joining and gap-filling, and clustered into unigenes. Finally, we obtained 46,999 unigenes, including 5,243 clusters and 41,756 singletons, with a mean length of 676 bp. The N50 of unigenes was 998 bp. The size distribution indicated that the lengths of the 9,114 unigenes were more than 1,000 bp (Figure 1).

**Table 1:** Summary for *A. c. cerana* transcriptome.

Total number of reads	55,303,332
Total clean Reads	51,581,510
Total clean Nucleotides (bp)	4,642,335,900
Q20 percentage	97.55%
GC percentage	41.92%
Total number of contigs	99,250
Mean length of contigs	332
Total number of unigenes	46,999
Mean length of unigenes	676
N50 of unigenes	998
Distinct clusters	5,243
Distinct singletons	41,756

### Functional annotation of unigenes

For annotation, all the distinct unigene sequences were searched against NR, Swissprot, GO, COG, and KEGG databases by BLASTX with a cut-off E-value of 10^−5^ (Table S1). By this method, a total of 24,630 unigenes (52.4% of all unigenes) returned an above cut-off BLAST result (Table 2). Of them, 24,001 unigenes were annotated by NCBI (51.07%), and 18,138 (38.59%), 15607 (33.21%), 7860 (16.72%), and 8064 (17.16%) unigenes by SwissProt, KEGG, COG, and GO respectively.

**Table 2:** Annotation of unigenes.

	Number of	Percentage of
Database	annotated unigenes	annotated unigenes
Nr	24,001	51.07%
Swissprot	18,138	38.59%
GO	8,064	17.16%
COG	7,860	16.72%
KEGG	15,607	33.21%
Total	24,630	52.41%

**Figure 2:**
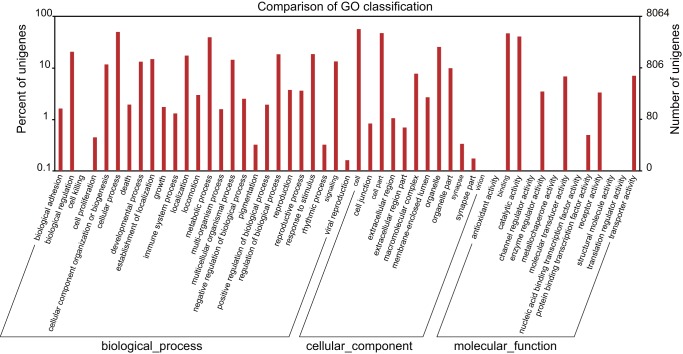
Gene Ontology classification of unigenes. Unigenes were annotated in three main categories: biological process, cellular component, and molecular function. The left y-axis indicates the percentage of a specific category of unigenes in that main category. The right y-axis indicates the number of unigenes in a category.

GO assignments were used to classify the functions of the predicted *A. c. cerana* unigenes. Based on sequence homology, 8,064 unigenes could be categorized into three main categories with a total of 51 functional groups (Figure 2). In each of the three main categories (biological process, cellular component, and molecular function) of the GO classification, “cellular process,” “cell,” and “binding” terms were dominant. We also noticed a high-percentage of genes in the categories of “metabolic process,” “cell part,” and “catalytic activity.”

**Figure 3:**
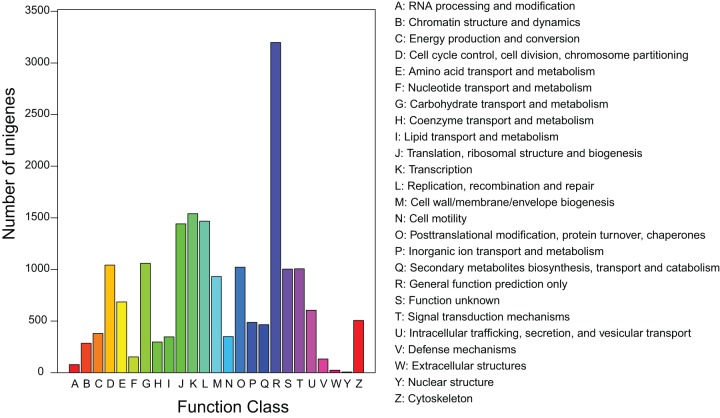
COG classification of unigenes. Out of 24,630 annotated unigenes, 7860 sequences had a COG classification among the 25 categories.

To further evaluate the function of the assembled unigenes, we searched the annotated sequences for the genes involved in Clusters of Orthologous Groups (COG). In total, out of 24,630 annotated unigenes, 7,860 unigenes had a COG classification (Figure 3). These unigenes were distributed in 25 COG categories, among them, the cluster “general function prediction” was the largest group (3,198), followed by “transcription” (1,540), “replication, recombination, and repair” (1,468) and “translation, ribosomal structure, and biogenesis” (1,442). The categories “nuclear structure (6),” “extracellular structures (24),” and “RNA processing and modification (78)” were the smallest groups.

The *A. c. cerana* unigenes were further annotated by mapping the 24,630 annotated sequences onto reference canonical pathways in the Kyoto Encyclopedia of Genes and Genomes (KEGG). In total, 15,607 sequences were assigned to 242 KEGG pathways (Table S2). The pathway in which unigenes were most enriched was “metabolic pathways” (1,882), followed by “regulation of actin cytoskeleton” (603), “pathways in cancer” (557), and “RNA transport” (542).

### Digital gene expression (DGE) library sequencing

**Figure 4:**
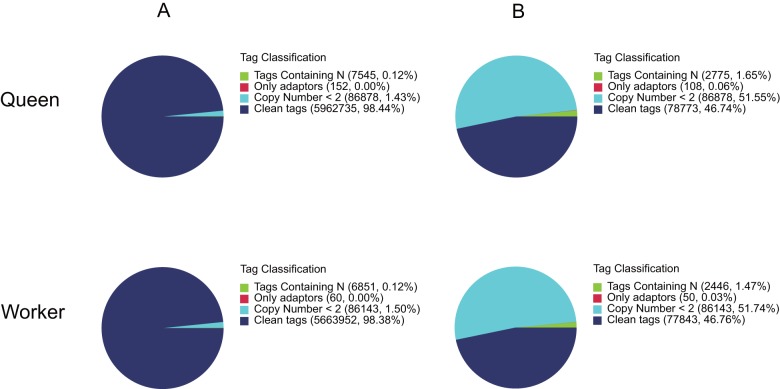
Distribution of total tags (A) and distinct tags (B) over different tag abundance categories in each sample. The numbers and percentage of tags containing N, empty tags with adaptor only, tags with copy number <2 and clean tags, are shown.

**Figure 5:**
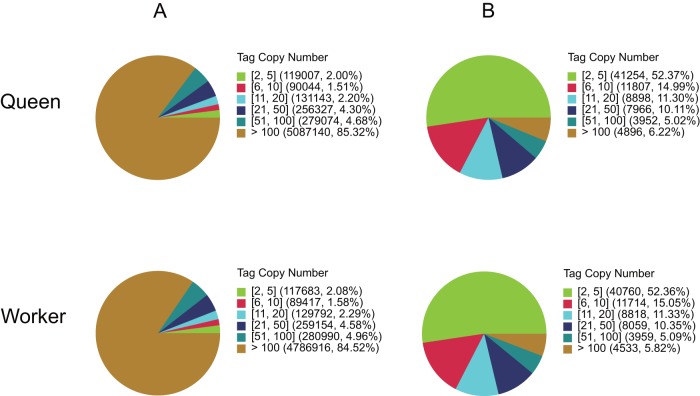
Distribution of total clean tags (A) and distinct clean tags (B) over different tag abundance categories in each sample. Numbers in the square brackets indicate the range of copy numbers for a specific category of tags. For example, [2], [5] means all the tags in this category has 2 to 5 copies. Numbers in the parentheses of (A) and (B) respectively show the total copy number of the clean tags and the total types of clean tags in that category.

*A. c. cerana* queen and worker DGE libraries were constructed and sequenced to investigate the expression profiles of all the unigenes between them, generating 6.06 and 5.76 million raw tags in each library. After filtering out low quality tags, the total number of clean tags in each library were 5.96 and 5.66 million (Table 3), and the percentage of clean tags relative to raw tags in each library were 98.44% and 98.38% (Figure 4). Among the clean tags, the number of sequences that could be mapped to reference unigenes were 3.77 and 3.96 million, and the percentage of these clean tags were 63.23% and 69.89% in the queen and worker libraries. In each library, clean tags with copy numbers of more than 100 were more than 84%, but their distribution in distinct clean tags did not exceed 7% (Figure 5). In contrast, the tags with copy numbers between 2 to 5 showed a broad distribution of distinct clean tags.

**Table 3:** Statistics of DGE sequencing.

Summary		Queen	Worker
Raw Data	Total	6057310	5757006
Raw Data	Distinct Tag	168534	166482
Clean Tag	Total number	5962735	5663952
Clean Tag	Distinct Tag number	78773	77843
All Tag Mapping to unigene	Total number	3770323	3958603
All Tag Mapping to unigene	Total % of clean tag	63.23%	69.89%
All Tag Mapping to unigene	Distinct Tag number	33185	33080
All Tag Mapping to unigene	Distinct Tag % of clean tag	42.13%	42.50%
Unambiguous Tag Mapping to unigene	Total number	3509889	3713507
Unambiguous Tag Mapping to unigene	Total % of clean tag	58.86%	65.56%
Unambiguous Tag Mapping to unigene	Distinct Tag number	28567	28490
Unambiguous Tag Mapping to unigene	Distinct Tag % of clean tag	36.26%	36.60%
All Tag-mapped unigenes	number	15214	15657
All Tag-mapped unigenes	% of ref unigenes	32.37%	33.31%
Unambiguous Tag-mapped unigenes	number	11399	11768
Unambiguous Tag-mapped unigenes	% of ref unigenes	24.25%	25.04%
Unknown Tag	Total number	2192412	1705349
Unknown Tag	Total % of clean tag	36.77%	30.11%
Unknown Tag	Distinct Tag number	45588	44763
Unknown Tag	Distinct Tag % of clean tag	57.87%	57.50%

Saturation analysis was performed to detect a positive association between the number of detected genes and the sequencing amount (total tag number). As shown in Figure S1, when the sequencing amount of the two DGE libraries reached near 3 M, the number of detected genes almost ceased to increase.

### Mapping tags to the reference transcriptome database

**Figure 6:**
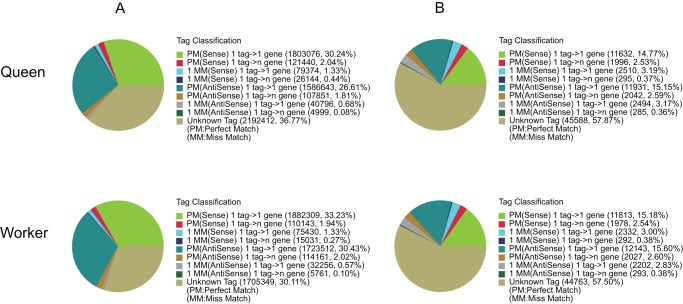
Distribution of total clean tags (A) and distinct clean tags (B) on unigenes. PM(Sense): perfect match to gene (sense); 1 tag->1 gene: one tag match to one gene; 1 tag->n gene: one tag match to more than one gene; 1 MM(Sense): match to gene (sense) with 1 bp mismatch; PM(AntiSense): perfect match to anti-sense gene; 1 MM(Anti-Sense): match to anti-sense gene with 1 bp mismatch; Unknown Tag: not match to gene (sense and anti-Sense).

To reveal the molecular events behind DGE profiles, we mapped the tag sequences of the two DGE libraries to our transcriptome reference database generated in the above mentioned Illumina sequencing. This reference database contained 46,999 distinct sequences with 59,762 unambiguous reference tags. Among the 78,773 and 77,843 distinct clean tags generated from the Illumina sequencing of the two libraries, 33,185 and 33,080 distinct clean tags were mapped to one or multiple unigenes in the reference database (Table 3). Tags mapped to a single unique sequence were the most critical subset of the DGE libraries, as they could explicitly identify a transcript. In the queen and worker libraries, 36.26% and 36.60% of distinct clean tags were mapped to unique sequences respectively. Of these, about half mapped to sense strand of the unigenes, and another half mapped to antisense strand of the unigenes (Figure 6). There were 36.77% and 30.11% of the total clean tags corresponding to 57.87% and 57.50% of the distinct clean tags could not be mapped to any unigene. Up to 24.25% (11,399) and 25.04% (11,768) of the unigenes in our transcriptome reference database could be unequivocally identified by unique tag (Table 3).

### Differentially expressed genes between queens and workers of *A. c. cerana*

To identify genes showing a significant change in expression between the newly emerged queens and workers, differentially expressed tags between these two libraries were identified by an algorithm developed by Audic et al [31]. Between worker and queen libraries, a total of 414 differentially expressed genes were detected, with 189 up-regulated genes and 225 down-regulated genes in queens (Table S3). Of the differentially expressed genes, 214 genes could not be annotated or annotated as “hypothetical protein” or “uncharacterized protein”; that is, their functions are unknown.

To understand the functions of these differentially expressed genes, all the differentially expressed genes were mapped to terms in the GO database and compared to the whole transcriptome background. Of the 414 differentially expressed genes, 72 genes have a GO ID and can be categorized into 172 functional groups in three main categories (Table S4). In each of the three main categories (biological process, cellular component, and molecular function) of the GO classification, “metabolic process,” “cell,” and “catalytic activity” terms were dominant. Moreover, two terms were significantly enriched (P-value <0.05) in the “biological process” category, while no terms were significantly enriched in the “cellular component” and “molecular function” categories. To further investigate the biochemical pathways of these differentially expressed genes, we mapped all of the differentially expressed genes to terms in KEGG database and compared this with the whole transcriptome background. Of the 414 differentially expressed genes, 157 unigenes had a KO ID and could be categorized into 140 pathways (Table S5). Of those, 16 pathways were significantly enriched (Q-value <0.05), and genes involved in metabolic pathways were the most significantly enriched.

In these differentially expressed genes, many were already reported to be differentially expressed between queens and workers. Vitellogenin (CL5161.Contig1), a critical protein involved in reproduction and caste differentiation [32], [33], and two hexamerin: hexamerin 110 (CL1143.Contig1) and hexamerin 70a (CL1653.Contig1), important factors reported to be involved in queen-worker caste differentiation of social insects [34]–[37], were all up-regulated in the queens. Other genes reported to be related to caste differentiation of the honey bee were also found in our study, including ribosomal proteins, cytochrome P450s, cuticle proteins, and odorant binding proteins [7]. We found six ribosomal protein genes (Unigene35563, Unigene2422, Unigene14816, Unigene7158, Unigene34160, and CL3917.Contig1) that were up-regulated in workers in comparison to the queens. Three cytochrome P450 genes (CL4487.Contig1, Unigene40252 and Unigene35287) were all up-regulated in queens. Five cuticle protein genes (Unigene16699, CL816.Contig1, CL782.Contig1, Unigene6752, Unigene15851) showed expression difference between queens and workers, with four (CL816.Contig1, CL782.Contig1, Unigene6752, Unigene15851) up-regulated in workers and one (Unigene16699) up-regulated in queens. We found two odorant binding proteins (Unigene15202, Unigene16694) both up-regulated in queens, and one pheromone-binding protein (Unigene15791) up-regulated in workers. Besides these, genes belonging to oxidoreductases, mitochondrial genes, and transporters also showed expression differences between queens and workers.

**Figure 7:**
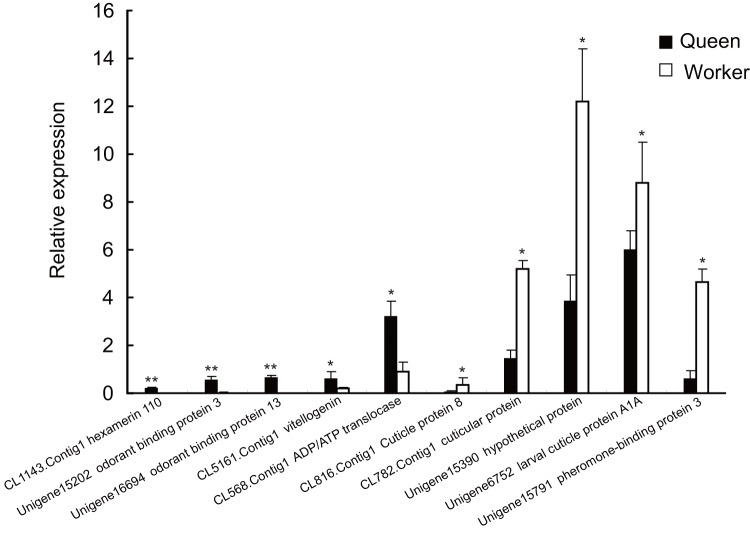
Verification of ten differentially expressed unigenes between queen and worker by quantitative RT-PCR. All ten unigenes showed significant difference (*p<0.05, **p<0.01) between queens and workers by *t*-test.

To verify the DGE data, we chose ten differentially expressed unigenes to detect their expression between adult queens and workers using quantitative RT-PCR (Table S6). Total RNAs from queens and workers were used as templates, and the *A. c. cerana β-actin* gene was chosen as the control. The result showed that the real time PCR results of all these unigenes were consistent with the DGE data (Figure 7).

## Discussion

Until recently, sequence data of *A. c. cerana* was scarce, so obtaining more sequence information was a priority for researchers in order to perform gene function research in *A. c. cerana*. In this study, we sequenced and annotated a reference transcriptome for the *A. c. cerana* using next-generation sequencing technologies, and obtained 4.64 Gb of transcriptome data and a total of 11.62 M of gene expression tags. As far as we know, this is the first study for obtaining whole transcriptome information using the RNA-seq approach in *A. c. cerana*. Our results provide the most extensive sequencing resource published for *A. c. cerana*.

In this study, we performed *de novo* assembly of transcriptome using short raw reads, due to a lack of *A. c. cerana* genome sequences. The mean length of the unigenes obtained in our study is 676 bp, which is significantly higher than in other studies using transcriptome sequencing and assembly [24], [25], [27]. Moreover, to further evaluate the sequencing and assembly quality, we downloaded 26 already reported *A. c. cerana* mRNA sequences containing the complete coding region from the Genbank, and searched for them in the *A. c. cerana* unigenes database using BLASTN with a cut-off E-value of 1e-30. The results showed a mean identity of 98.0% and query coverage of 82.06%, suggesting high quality of sequencing and assembly.

In the genus *Apis*, *A. cerana* and *A. mellifera* have the closest evolutionary relationship [38], so genetic divergence between them should be slight, and they should share most of their functional genes. The whole genome sequence of *A. mellifera* is available and all of the function genes are predicted, which allows us to compare the *A. c. cerana* unigenes in this study with the predicted genes of *A. mellifera*. We aligned all of the unigenes with all the *A. mellifera* genes (including alternative splicing variants), downloaded from NCBI (ftp://ftp.ncbi.nih.gov/genomes/Apis_mellifera/RNA/rna.fa.gz), using BLASTN with an E-value of 1e-30, and found that 10,104 genes (86.09%) of all the predicted *A. mellifera* 11,736 genes can be matched to the *A. c. cerana* unigenes. These results indirectly suggest that our transcriptome sequences have a good coverage for all the *A. c. cerana* genes. We further analyzed the differences of genes related to important physiological characteristics between these two species, and found that the number of odorant receptor genes in *A. c. cerana* is far less than the number reported in *A. mellifera* [39]. There are just 18 unigenes annotated as “odorant receptor”, compared to 170 in *A. mellifera* [39]. One possible reason is that these odorant receptor genes are specifically expressed in the antennae of honey bees [39]. Because RNAs from the antennae just occupy a very small proportion of the total RNA, their abundance might be much lower compared to those from other tissues. However, this explanation requires experimental verification.

Through DGE analysis, we obtained a total of 414 differentially expressed genes between queens and workers of *A. c. cerana*. Many of them were reported to be caste-specifically-expressed in previous research, such as vitellogenin [32], [33] and hexamerins [34]–[37], which are key factors involved in the caste differentiation. Vitellogenin is reported to have antioxidant functions and thereby can prolong lifespan of the reproductive queen castes [33], while acting as a hormone to affect the future foraging behavior of workers [32]. Hexamerins, on the other hand, have been shown to regulate the soldier-caste differentiation in the termite *Reticulitermes flavipes* [36], [37]. These results not only suggest that the results of our DGE analysis are reliable, but also indicate that DGE is an efficient method to identify caste related genes in the honey bee.

To detect whether these differentially expressed genes between queens and workers are conserved among species, we compared all the differentially expressed genes detected in our study to those reported in other Hymenoptera insects, including bumblebee *Bombus terrestris* [20], stingless bee *Melipona quadrifasciata* [21], [22], the ant *Lasius niger* [23] and *A. mellifera* [5]–[7]. We found that just a few differentially expressed genes detected in the first three species overlapped with our results: a hexamerin gene, a cuticle protein gene, and a 60S ribosomal protein gene in *Bombus terrestris*, a cytochrome P450 gene in *Melipona quadrifasciata* and a vitellogenin gene in *Lasius niger*. The lack of overlap may be due to the relatively small number of differentially expressed genes detected in the other three species, due to the limitation of experimental methods adopted in these studies. When we compared the differentially expressed genes in our study to those in *A. mellifera* reported by three different research groups [5]–[7], 73 of the 414 DGE detected in our study overlapped between these two species, including viotellogenin, hexamerins, crystallin, cytochrome P450 genes, ribosomal protein genes, and cuticle protein genes. There might be more DGEs overlap between the *A. c. cerana* and *A. mellifera*, but were missed because the *A. mellifera* studies used whole body larvae or adults heads, while our study used whole body adults. All these results suggest that the pathway of caste differentiation of these social insects maybe have some degree of conservation among species.

Secreting royal jelly is an important feature of workers, and one of the most important differences between workers and queens. In this study, 7 ribosomal protein genes, which code for the main component of the ribosome and play important roles in protein synthesis, were up-regulated in workers. This may be due to the fact that the workers need to synthesize royal jelly, which is mainly composed of royal jelly proteins, while the queen does not. Although the sampled workers were newly emerged adult workers, at this stage the workers may have already begun to up-regulate the expression of these ribosomal protein genes to promote royal jelly protein synthesis.

Besides the reported genes related to the caste-specific differentiation of queens and workers in *A. mellifera* or other Hymenopteran insects, we also found many other genes showing expression differences between queens and workers. For example, four “venom” related genes (CL3467.Contig1, CL415.Contig1, Unigene6964, and Unigene7666) were up-regulated in queens. Interestingly, we found that two circadian rhythm genes, *period* (CL4008.Contig1) and *takeout* (Unigene15755) were both up-regulated in workers. Workers may have a stronger circadian rhythm than the queens because the queens usually lay eggs around the clock [40], while workers eventually venture outside to forage during the day time and rest at night.

In addition to those functionally annotated genes, there were also 214 differentially expressed genes with unknown functions, which may be involved in the queen-worker caste determination or caste-specific differentiation of many physiological characteristics. Although their functions remained unknown, some of them may be critical in differentiating between the queens and the workers.

In summary, we obtained whole transcriptome sequences of the *A. c. cerana* by high-throughput sequencing, and analyzed the gene expression differences between queens and workers. These results will provide a solid foundation for research on the molecular mechanisms of the biological traits of *A. c. cerana*.

## Conclusions

Through next generation high throughput sequencing we obtained 46,999 unique sequences. Based on a similarity search with known proteins, a total of 24,630 unigenes were identified to have BLAST hits with a cut-off E-value above 10^−5^. Using these assembled sequences as a reference, we compared the gene expression differences between the adult queens and workers of *A. c. cerana*. A total of 414 genes showed differential expression between them. From these genes, we found many genes related to the physiological differences between the queens and workers. Our results will provide invaluable clues for researching the physiological differences between queens and workers.

## Materials and Methods

### Insect

*A. c. cerana* were sampled from Honey bee Research Institute, Jiangxi Agricultural University, China. For transcriptome analysis three day old worker larvae; one day old worker pupae; and one day old adult workers, foragers, and nurses were sampled with five individuals per group. Total RNAs from these samples were isolated and pooled as one sample for transcriptome sequencing to obtain more sequence information of all the transcribed genes in *A. c. cerana*. For DGE analysis, five newly emerged workers and queens were sampled as two samples for RNA isolation and sequencing. The reason for choosing newly emerged adult stage in our DGE analysis is that this stage represents the endpoint of postembryonic development and the starting point for caste-specific task performance [3], and it is an important stage for exploring the molecular mechanism of many caste-specific traits of honeybee.

### cDNA library preparation and Illumina sequencing for transcriptome analysis

To construct a cDNA library, total RNA was extracted from the above mentioned five *A. c. cerana* samples using the SV total RNA isolation system (Promega, USA) according to the manufacturer's protocol. Then, these five RNA samples were pooled (with equal amount of RNA from each stage) as one sample for transcriptome sequencing to obtain as much gene expression information as possible. Poly(A)+RNA was isolated from 20 µg of pooled total RNA using oligo(dT) magnetic beads according to Illumina manufacturer's instructions. Then, the purified mRNA was fragmented into short sequences in the fragmentation buffer at 94°C for 5 min. These cleaved, short RNA sequences were used as templates for first strand cDNA synthesis primed with random hexamers. The second-strand cDNA was synthesized using buffer, dNTPs, RNase H, and DNA polymerase I. Short fragments were purified with a QiaQuick PCR extraction kit (Qiagen, Germany) and resolved with EB buffer for end repair and poly(A) addition. Then, sequencing adaptors were added to the fragments, and suitable fragments were used as templates for PCR amplification after agarose gel electrophoresis, PCR products were purified using the QiaQuick PCR extraction kit (Qiagen, Germany) to create a cDNA library. Finally, the library (200 bp insert) was sequenced from both 5′ and 3′ ends using Illumina HiSeq TM 2000 (Illumina Inc., SanDiego, CA, USA). Sequencing-received raw image data was transformed by base calling into sequence data, which is called raw data or raw reads, and was stored in fastq format.

### Analysis of Illumina sequencing results

Before performing bioinformatical analysis, the raw sequences were filtered to remove low quality reads. The filtration steps were as follows: 1, remove reads just containing adaptor sequence; 2, remove reads containing unknown nucleotide “N” over 5%; and 3, remove low quality reads (those with a ratio of bases with a quality value lower than 10 occupying more than 20% of the whole read). The remaining clean reads were used for further analysis and were deposited in the NCBI sequence read archive (SRX175819).

Transcriptome *de novo* assembly was carried out with a short reads assembling program–Trinity [41]. The Trinity software first combined reads with a certain length of overlap to form longer fragments without N, forming contigs. Then the reads were mapped back to contigs, with paired-end reads it was able to detect contigs from the same transcript as well as the distances between these contigs. Then, Trinity connected these contigs to get consensus sequences that contained the least Ns and could not be extended on either end. Such sequences were defined as unigenes.

Finally, the obtained unigenes were searched against the protein databases NR, Swiss-Prot, KEGG, and COG using BLASTX with E-value <10^−5^ to decide their direction. The best matched hits were used to decide the sequence direction of each unigene. If the search results of different databases conflicted with each other, a priority order of NR, Swiss-Prot, KEGG, and COG was followed. If a unigene could not be aligned to any of the above four databases, the software ESTScan [42] was introduced to decide its sequence direction.

After assembly, all the unigenes were firstly aligned by BLASTX to protein databases NR, Swiss-Prot, KEGG and COG (E-value <10^−5^), retrieving proteins with the highest sequence similarity with the given unigenes along with their protein functional annotations. Based on NR annotation, we used the Blast2GO program [43] to get the GO annotations of unigenes. After getting GO annotation for each unigene, we used WEGO software [44] to do GO functional classification for all unigenes and to understand the distribution of gene functions of the species from the macro level.

### Digital gene expression library preparation and sequencing

To construct the DGE library, total RNA was extracted from the one day old queen and worker samples using the SV Total RNA isolation System (Promega, USA) according to the manufacturer's protocol. Then, the libraries were constructed using the Illumina gene expression sample prep kit according to its protocol. Briefly, poly(A)+ RNA was purified from 6 µg of total RNA, using oligo(dT) magnetic beads. First, strand cDNA were directly synthesized on the poly(A)+ RNA-bound beads primed by oligo(dT). Then, the second strand was synthesized and digested with NlaIII, which recognized the CATG site. The digested cDNA fragments containing the 3′ ends were purified from the magnetic beads, and then the Illumina adaptor1 was added to the 5′ ends of these cDNA fragments. These fragments were further digested by another endonuclease MmeI, which recognized the junction of the Illumina adaptor1 and the CATG site, and cut at 17 bp downstream of CATG site to produce 21 bp tags containing the adaptor1 sequence. After removing the cleaved 3′ end sequences with magnetic beads precipitation, the Illumina adaptor2 was ligated to the 3′ ends of the tags to create a tag library containing many tags with different adaptors on both ends. Then, the library was amplified by PCR for 15 cycles. PCR products were segregated on 6% PAGE gel electrophoresis, and the 105 bp fragments were chosen and purified for sequencing. The double-strand DNA fragments were denatured, and the single-stranded molecules were fixed onto the Illumina sequencing chip for sequencing. Each tunnel of chip (flowcell) generated millions of raw tags with a length of 49 bp.

### Analysis and mapping of DGE tags to transcriptome sequences

Sequencing-received raw image data was transformed by base calling into sequence data and stored in fastq format. Raw sequences were filtered by the following steps: 1, remove adaptor sequence (since tags are only 21 nt long while the sequencing reads are 49 nt long, raw sequences are with 3′ adaptor sequences); 2, remove empty tags (no tag sequence between the adaptors); 3, remove low quality tags (tags with unknown nucleotide “N”); 4, remove tags with only one copy number (which might result from sequencing errors); 5, remove of tags which are too long or too short. After filtration, the remained clean tags contain CATG and 21 bp tag sequences, and were deposited in the NCBI sequence read archive (SRX175818 for queens and SRX180263 for workers).

Before mapping, a tag library containing all the possible CATG+17 nt tag sequences was created using the assembled *A. c. cerana* transcriptome sequence data. Then, all the clean tags were mapped to this transcriptome reference database with only one nucleotide mismatch allowed. Clean tags that mapped to multiple genes were filtered. The remaining clean tags were designated as unambiguous clean tags. For gene expression analysis, the number of unambiguous clean tags for each gene was calculated and normalized to TPM (number of transcripts per million clean tags).

### Evaluation of DGE libraries

To identify the differentially expressed genes between queen and worker libraries, a rigorous statistical algorithm was developed by consulting the method described by Audic [31] to statistically analyze the tag frequency in each DGE library. The false discovery rate (FDR) was used to determine the threshold P-value in multiple tests. A FDR<0.001 and an absolute E-value of the log2 ratio>1 were used as the threshold to determine significant differences in gene expression. The identified differentially expressed genes were used for GO and KO enrichment analysis.

GO enrichment analysis of functional significance applies a hypergeometric test to map all differentially expressed genes to terms in GO database, looking for significantly enriched GO terms in differentially expressed genes comparing to the genome background. The calculating formula is:
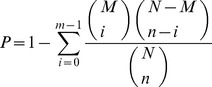
Where *N* is the number of all genes with GO annotation; *n* is the number of differentially expressed genes in *N*; *M* is the number of all genes that are annotated to the certain GO terms; *m* is the number of differentially expressed genes in *M*.

KEGG pathway enrichment analysis identifies significantly enriched metabolic pathways or signal transduction pathways in differentially expressed genes comparing with the whole genome background. The calculating formula is the same as that of GO analysis.

### Quantitative RT-PCR validation

Total RNA was extracted from one day old queens and workers as described for the DGE library preparation. RNA integrity was determined by agarose gel (1%), electrophoresis, and ethidium bromide staining. The purity (260 nm/280 nm ratio between 1.8 and 2.0 for RNA) and concentration of each RNA sample was measured in triplicate using a UV spectrophotometer (GeneQuant, Pharmacia). The RNA sample was standardized to 1 μg/μl for reverse transcription. cDNA was synthesized using MLV reverse transcriptase (Takara, Japan) according to the manufacturer's instructions, and *β-actin* was used as an internal control [45]. qPCR primers were designed based on the nucleotide sequence of the chosen unigenes using Primer 5.0 software. Primer sequences and unigenes are summarized in Additional File 7.

The cycling conditions were as follows: preliminary 94°C for 2 min, 40 cycles including 94°C for 15 sec, 63°C for 30 sec, and 72°C for 30 sec. The specificity of the PCR products was verified by melting curve analysis for each sample. For each unigene, five biological replicates (with three technical replicate for each biological replicate) were performed. The control and target unigene for each sample were run in the same plate to eliminate interplate variation. The Ct value for each biological replicate was obtained by calculating the arithmetic mean of three technical replicate values. The relative expression level between queens and workers was calculated using the formula reported by Liu and Saint's [46]. The differential expression was analyzed by analysis of variance (ANOVA) using StatView 5.01 (SAS Institute, Gary, NC, USA).

## Supporting Information

Figure S1 **Saturation analysis of clean tags.** With the increase of total sequence number, the number of detected genes gradually ceased to increase.
(EPS)

Table S1 Annotation of all the unigenes. 24,630 of the 46999 unigenes were annotated using BLASTX search in the five public databases (NR, Swissport, GO, COG, KEGG) with a cut-off E-value of 10^−5^.
(RAR)

Table S2 KEGG analysis of the 15607 unigenes.
(XLS)

Table S3 The differentially expressed genes between queens and workers. TPM: transcript copies per million tags. Raw intensity: the total number of tags sequenced for each gene. FDR: false discovery rate. We used FDR ≤0.001 and the absolute value of log_2_Ratio ≥1 as the threshold to judge the significance of gene expression difference. In order to calculate the log_2_Ratio and FDR, we used TPM value of 0.01 instead of 0 for genes that do not express in one sample.
(XLS)

Table S4 Gene Ontology enrichment analysis of the differentially expressed genes. The results were summarized in three main categories: biological process, cellular component and molecular function.
(XLS)

Table S5 KEGG pathway enrichment analysis of the differentially expressed genes.
(XLS)

Table S6 Primers used for quantitative RT-PCR analysis.
(DOC)

## References

[1] Peng YS, Fang ZY, Xu SY, Ge LS (1987) The resistance mechanism of the Asian honey bee, *Apis cerana cerana* Fabr., to ectoparasitic mite, *Varroa jacbsoni* Oudemans. J Inver Patho 47: 54–60.

[2] Cheng SL (2001) Special management of *Apis cerana cerana*. In: Liu BH, editor. The Apicultural Science in China. Beijing: Chinese Agricultural Press. 488–512.

[3] Page RE Jr, Peng CY (2001) Aging and development in social insects with emphasis on the honey bee, *Apis mellifera L*. Experimental Gerontology. 36(4–6): 695–711. 10.1016/s0531-5565(00)00236-9 11295509

[4] Severson DW, Williamson JL, Aiken JM (1989) Caste-specific transcription in the female honey bee. Insect Biochem 19(2): 215–220.

[5] Evans JD, Wheeler DE (1999) Differential gene expression between developing queens and workers in the honey bee, *Apis mellifera* . Proc Natl Acad Sci U S A 96(10): 5575–5580. 10318926 10.1073/pnas.96.10.5575 PMC21902

[6] Evans JD, Wheeler DE (2001) Expression profiles during honeybee caste determination. Genome Biol 2(1): RESEARCH0001. 11178278 10.1186/gb-2000-2-1-research0001 PMC17597

[7] Barchuk AR, Cristino AS, Kucharski R, Costa LF, Simões ZL, et al (2007) Molecular determinants of caste differentiation in the highly eusocial honeybee *Apis mellifera* . BMC Dev Biol 7: 70. 17577409 10.1186/1471-213X-7-70 PMC1929063

[8] Azevedo SV, Caranton OA, de Oliveira TL, Hartfelder K (2011) Differential expression of hypoxia pathway genes in honey bee (*Apis mellifera L*.) caste development. J Insect Physiol 57(1): 38–45. 20887729 10.1016/j.jinsphys.2010.09.004

[9] Patel A, Fondrk MK, Kaftanoglu O, Emore C, Hunt G, et al (2007) The making of a queen: TOR pathway is a key player in diphenic caste development. PLoS One 2(6): e509. 17551589 10.1371/journal.pone.0000509 PMC1876819

[10] Mutti NS, Dolezal AG, Wolschin F, Mutti JS, Gill KS, et al (2011) IRS and TOR nutrient-signaling pathways act via juvenile hormone to influence honey bee caste fate. J Exp Biol 214(Pt 23): 3977–3984. 10.1242/jeb.061499 PMC3212421 22071189

[11] Wheeler DE, Buck N, Evans JD (2006) Expression of insulin pathway genes during the period of caste determination in the honey bee, *Apis mellifera* . Insect Mol Biol 15(5): 597–602. 17069635 10.1111/j.1365-2583.2006.00681.x PMC1761130

[12] de Azevedo SV, Hartfelder K (2008) The insulin signaling pathway in honey bee (*Apis mellifera*) caste development – differential expression of insulin-like peptides and insulin receptors in queen and worker larvae. J Insect Physiol 54(6): 1064–1071. 18513739 10.1016/j.jinsphys.2008.04.009

[13] Corona M, Hughes KA, Weaver DB, Robinson GE (2005) Gene expression patterns associated with queen honey bee longevity. Mech Ageing Dev 126(11): 1230–1238. 16139867 10.1016/j.mad.2005.07.004

[14] Grozinger CM, Fan Y, Hoover SE, Winston ML (2007) Genome-wide analysis reveals differences in brain gene expression patterns associated with caste and reproductive status in honey bees (*Apis mellifera*). Mol Ecol 16(22): 4837–4848. 17927707 10.1111/j.1365-294X.2007.03545.x

[15] Humann FC, Hartfelder K (2011) Representational Difference Analysis (RDA) reveals differential expression of conserved as well as novel genes during caste-specific development of the honey bee (*Apis mellifera L*.) ovary. Insect Biochem Mol Biol 41(8): 602–612. 21477651 10.1016/j.ibmb.2011.03.013

[16] Thompson GJ, Yockey H, Lim J, Oldroyd BP (2007) Experimental manipulation of ovary activation and gene expression in honey bee (*Apis mellifera*) queens and workers: testing hypotheses of reproductive regulation. J Exp Zool A Ecol Genet Physiol 307(10): 600–610. 17786975 10.1002/jez.415

[17] Li J, Wu J, Begna Rundassa D, Song F, et al (2010) Differential protein expression in honeybee (*Apis mellifera L*.) larvae: underlying caste differentiation. PLoS One 5(10): e13455. 20975997 10.1371/journal.pone.0013455 PMC2958119

[18] Begna D, Fang Y, Feng M, Li J (2011) Mitochondrial proteins differential expression during honeybee (*Apis mellifera L*.) queen and worker larvae caste determination. J Proteome Res 10(9): 4263–4280. 21751814 10.1021/pr200473a

[19] Begna D, Han B, Feng M, Fang Y, Li J (2012) Differential Expressions of Nuclear Proteomes between Honeybee (*Apis mellifera L*.) Queen and Worker Larvae: A Deep Insight into Caste Pathway Decisions. J Proteome Res 11(2): 1317–1329. 22200504 10.1021/pr200974a

[20] Pereboom JJ, Jordan WC, Sumner S, Hammond RL, Bourke AF (2005) Differential gene expression in queen-worker caste determination in bumble-bees. Proc Biol Sci 272(1568): 1145–1152. 16024376 10.1098/rspb.2005.3060 PMC1559810

[21] Judice C, Hartfelder K, Pereira GAG (2004) Caste-specific gene expression in the stingless bee *Melipona quadrifasciata* – Are there common patterns in highly eusocial bees? Insect Soc 51(4): 352–358.

[22] Judice CC, Carazzole MF, Festa F, Sogayar MC, Hartfelder K, et al (2006) Gene expression profiles underlying alternative caste phenotypes in a highly eusocial bee, *Melipona quadrifasciata* . Insect Mol Biol 15(1): 33–44. 16469066 10.1111/j.1365-2583.2005.00605.x

[23] Gräff J, Jemielity S, Parker JD, Parker KM, Keller L (2007) Differential gene expression between adult queens and workers in the ant *Lasius niger* . Mol Ecol 16(3): 675–683. 17257122 10.1111/j.1365-294X.2007.03162.x

[24] Wang XW, Luan JB, Li JM, Bao YY, Zhang CX, et al (2010) De novo characterization of a whitefly transcriptome and analysis of its gene expression during development. BMC Genomics 11: 400. 20573269 10.1186/1471-2164-11-400 PMC2898760

[25] Xue J, Bao YY, Li BL, Cheng YB, Peng ZY, et al (2010) Transcriptome analysis of the brown planthopper *Nilaparvata lugens* . PLoS One 5(12): e14233. 21151909 10.1371/journal.pone.0014233 PMC2997790

[26] Hao DC, Ge G, Xiao P, Zhang Y, Yang L (2011) The first insight into the tissue specific taxus transcriptome via Illumina second generation sequencing. PLoS One 6(6): e21220. 21731678 10.1371/journal.pone.0021220 PMC3120849

[27] Xia Z, Xu H, Zhai J, Li D, Luo H, et al (2011) RNA-Seq analysis and de novo transcriptome assembly of *Hevea brasiliensis* . Plant Mol Biol 77(3): 299–308. 21811850 10.1007/s11103-011-9811-z

[28] Markovets AA, Herman D (2011) Analysis of cancer metabolism with high-throughput technologies. BMC Bioinformatics (Suppl 10):S8. 10.1186/1471-2105-12-S10-S8 PMC3236851 22166000

[29] Trapnell C, Williams BA, Pertea G, Mortazavi A, Kwan G, et al (2010) Transcript assembly and quantification by RNA-Seq reveals unannotated transcripts and isoform switching during cell differentiation. Nat Biotechnol 28(5): 511–515. 20436464 10.1038/nbt.1621 PMC3146043

[30] Mu Y, Ding F, Cui P, Ao J, Hu S, et al (2010) Transcriptome and expression profiling analysis revealed changes of multiple signaling pathways involved in immunity in the large yellow croaker during Aeromonas hydrophila infection. BMC Genomics 11: 506. 20858287 10.1186/1471-2164-11-506 PMC2997002

[31] Audic S, Claverie JM (1997) The significance of digital gene expression profiles. Genome Res 7(10): 986–995. 9331369 10.1101/gr.7.10.986

[32] Guidugli KR, Nascimento AM, Amdam GV, Barchuk AR, Omholt S, et al (2005) Vitellogenin regulates hormonal dynamics in the worker caste of a eusocial insect. FEBS Lett 579(22): 4961–4965. 16122739 10.1016/j.febslet.2005.07.085

[33] Corona M, Velarde RA, Remolina S, Moran-Lauter A, Wang Y, et al (2007) Vitellogenin, juvenile hormone, insulin signaling, and queen honey bee longevity. Proc Natl Acad Sci U S A 104(17): 7128–7133. 17438290 10.1073/pnas.0701909104 PMC1852330

[34] Martins JR, Nunes FM, Cristino AS, Simões ZL, Bitondi MM (2010) The four hexamerin genes in the honey bee: structure, molecular evolution and function deduced from expression patterns in queens, workers and drones. BMC Mol Biol 11: 23. 20346164 10.1186/1471-2199-11-23 PMC2861669

[35] Martins JR, Anhezini L, Dallacqua RP, Simões ZL, Bitondi MM (2011) A honey bee hexamerin, HEX 70a, is likely to play an intranuclear role in developing and mature ovarioles and testioles. PLoS One 6(12): e29006. 22205988 10.1371/journal.pone.0029006 PMC3242770

[36] Zhou X, Oi FM, Scharf ME (2006) Social exploitation of hexamerin: RNAi reveals a major caste-regulatory factor in termites. Proc Natl Acad Sci U S A 103(12): 4499–4504. 16537425 10.1073/pnas.0508866103 PMC1450200

[37] Zhou X, Tarver MR, Bennett GW, Oi FM, Scharf ME (2006) Two hexamerin genes from the termite *Reticulitermes flavipes*: Sequence, expression, and proposed functions in caste regulation. Gene 376(1): 47–58. 16580793 10.1016/j.gene.2006.02.002

[38] Willis LG, Winston ML, Honda BM (1992) Phylogenetic relationships in the honeybee (genus Apis) as determined by the sequence of the cytochrome oxidase II region of mitochondrial DNA. Mol Phylogenet Evol 1(3): 169–178. 1342933 10.1016/1055-7903(92)90013-7

[39] Robertson HM, Wanner KW (2006) The chemoreceptor superfamily in the honey bee, *Apis mellifera*: expansion of the odorant, but not gustatory, receptor family. Genome Res 16: 1395–1403. 17065611 10.1101/gr.5057506 PMC1626641

[40] Zeng ZJ (2009) The biology of the honeybee. In: Wu XF, editor. Apiculture. Beijing: Chinese Agricultural Press. 36–38.

[41] Grabherr MG, Haas BJ, Yassour M, Levin JZ, Thompson DA, et al (2011) Full-length transcriptome assembly from RNA-Seq data without a reference genome. Nat Biotechnol 29(7): 644–652. 21572440 10.1038/nbt.1883 PMC3571712

[42] Iseli C, Jongeneel CV, Bucher P (1999) ESTScan: a program for detecting, evaluating, and reconstructing potential coding regions in EST sequences. Proc Int Conf Intell Syst Mol Biol 138–148. 10786296

[43] Conesa A, Götz S, García-Gómez JM, Terol J, Talón M, et al (2005) Blast2GO: a universal tool for annotation, visualization and analysis in functional genomics research. Bioinformatics 21(18): 3674–3676. 16081474 10.1093/bioinformatics/bti610

[44] Ye J, Fang L, Zheng H, Zhang Y, Chen J, et al.. (2006) WEGO: a web tool for plotting GO annotations. Nucleic Acids Res 34(Web Server issue):W293–297. 10.1093/nar/gkl031 PMC1538768 16845012

[45] Lourenço AP, Mackert A, Cristino AS, Simões ZLP (2008) Validation of reference genes for gene expression studies in the honey bee, *Apis mellifera*, by quantitative real-time RT-PCR. Apidologie 39(3): 372–385.

[46] Liu W, Saint DA (2002) A new quantitative method of real time reverse transcription polymerase chain reaction assay based on simulation of polymerase chain reaction kinetics. Anal Biochem 302(1): 52–59. 11846375 10.1006/abio.2001.5530

