# Supplementary material for: Transcriptome Analysis of the Asian Honey Bee Apis cerana cerana
Source: PLoS One. 2012 Oct 24;7(10):e47954. doi: 10.1371/journal.pone.0047954 (PMC3480438; doi:10.1371/journal.pone.0047954)
Supplement: Table S6 — Primers used for quantitative RT-PCR analysis. (DOC) [file pone.0047954.s007.doc]

Table S6 Primers used for quantitative RT-PCR analysis

| unigene | primers |
| --- | --- |
| CL1143.Contig1 | 5' CTGGCGGCTGGCAAAGTATC 3'  5' CTTGGTGGAAGACGAGAACATC 3' |
| unigene15202 | 5' CCGTTCATCAGAGCCATCC 3'  5' TGTTCGCATTTCCAAGTTTC 3' |
| unigene16694 | 5' TTTGTCTCGTTGGTATTCTG 3'  5' CGTCTGCTTTCTGTAGGTC 3' |
| CL5161.Contig1 | 5' CACGCTCCTCAGGCTCAAC 3'  5' CACGCATCACGAATACGACTA 3' |
| CL568.Contig1 | 5' GTGGTGCTGCTGGTGCTACA 3'  5' TGATGATACCCTGGACGGATAC 3' |
| CL816.Contig1 | 5' CACTTCTAACGCTTCTTCTTGT 3'  5' GTTCTTCCACGCTACCCTTC 3' |
| CL782.Contig1 | 5' CATCCTTCCTCCCTTCTCTTG 3'  5' GAACATCTCACCGTCCATCG 3' |
| unigene15390 | 5' CTGCTGGCGACAAGAGGACC 3'  5' GCCCACATCAACGAAAGAATCAA 3' |
| unigene6752 | 5' ATGCTGTTGTTCAGAAAGACCCT 3'  5' GCTGTGGCGATAACCGATGT 3' |
| unigene15791 | 5' TCATTGTGGGACACGACCGA 3'  5' CATTGACCATTCCTTACTGCCTCT 3' |
